# Professor Lombroso and Vacher’s Brain

**Published:** 1899-09

**Authors:** 


### *EDITORIAL NOTES AND COMMENTS.*

---

The Business office of THE JOURNAL-RECORD is 305, 306 Fitten Building.

The Editorial office is 318-319-320 The Prudential.

Address all Business communications to Dr. M. B. Hutchins, Mgr.

Make remittances payable to THE ATLANTA JOURNAL-RECORD OF MEDICINE.

On matters pertaining to the Editorial and Original Communications address Dr. Bernard Wolff, Atlanta.

Reprints of original articles will be furnished at cost price. Requests for the same should always be made *on the manuscript*.

We will present, post-paid, on request, to each contributor of an original article, twenty (20) marked copies of THE JOURNAL-RECORD containing such article.

---

### PROFESSOR LOMBROSO AND VACHER'S BRAIN.

PROF. CESARE LOMBROSO has done much for criminal anthropology. More than any one else, he has striven to reduce the subject to something like system. But for some time those interested in this study have been inclined to listen to Lombroso with one eye closed and the tongue in the cheek. This was noticeably the case at the recent Congress of Criminal Anthropologists at which he sought to revive interest in his conception of the congenital criminal. In a late number of the *Revue Scientifique* he gives his opinion on the brain of Vacher, the multi-murderer of the Jack-the-Ripper type, who was executed for his crimes. After having examined an impression of his brain Lombroso declares that he found there all the characteristic anomalies of the epileptic and the congenital criminal, and proceeds to enumerate them. Now in the issue of *Le Progrès Médical* for August 5, the critic is himself criticized by G. Paul Boncour who says: "In the first place, I may remark that M. Lombroso never saw the brain. *He only saw one impression.* I have had this impression in my hands, having been shown it many times by M. Manouvrier at the same time that it was shown to many others, and I declare with all those who examined it that it is impossible to detect in the cast any defect or any of the peculiarities enumerated to the *Revue Scientifique.* In the second place, supposing that Lombroso did what no one else could and distinguished something in the model, it seems to me that this fact is of no value as the observation was made on the left hemisphere, and an exact design of the entire brain could not be reproduced from this. If circumstances permitted I would reproduce here the cast of the left hemisphere and every one could form his own judgment. However, it is easy to refer to the communication on Vacher made to the Anthropological Society by Drs. Laborde and Manouvrier. The original sections of the brain were there presented, and the members of the Society could judge for themselves, and none of the peculiarities claimed by Lombroso was found to exist. Such ill-founded assertions should not be allowed to pass in a dignified and serious scientific journal without challenge, especially when they tend to throw discredit upon judges, experts, and juries. This has determined me to return once more to a case which has already wearied the press. It was Lombroso who began it, and my excuse is therefore good."

Lombroso may have something to say as a rejoinder, but it appears that he subjected the material in the case to somewhat Procrustean methods to adjust it to his theories.

